# Assessment of 1183 screen-detected, category 3B, circumscribed masses by cytology and core biopsy with long-term follow up data

**DOI:** 10.1038/sj.bjc.6604296

**Published:** 2008-04-01

**Authors:** G Farshid, P Downey, P G Gill, S Pieterse

**Affiliations:** 1 BreastScreen SA, 1 Goodwood Road, Wayville 5065, South Australia

**Keywords:** biopsy, breast, mammography, screening

## Abstract

Discrete masses are commonly detected during mammographic screening and most such lesions are benign. For lesions without pathognomonically benign imaging features that are still regarded likely to be non-malignant (Tabar grade 3) reliable biopsy results would be a clinically useful alternative to mammographic surveillance. Appropriate institutional guidelines for ethical research were followed. Between Jan 1996–Dec 2005 grade 3B discrete masses detected in the setting of a large, population based, breast cancer screening programme are included. Patient demographics, fine needle aspiration biopsy (FNAB), core and surgical biopsy results are tabulated. The final pathology of excised lesions was obtained. Information regarding interval cancers was obtained from the State Cancer Registry records and also through long term follow-up of clients in subsequent rounds of screening. A total of 1183 lesions, mean diameter of 13.3 mm (±8.3 mm) and mean client age of 55.1 years (±8.8 years) are included. After diagnostic work up, 98 lesions (8.3%) were malignant, 1083 were non-malignant and a final histologic diagnosis was not established in two lesions. In the 27 months after assessment, no interval cancers were attributable to these lesions and during a mean follow up of 54.5 months, available in 84.9% of eligible women, only one cancer has developed in the same quadrant as the original lesion, although the two processes are believed to be unrelated. FNAB performed in 1149 cases was definitive in 80.5% cases (882 benign, 43 malignant) with a negative predictive value (NPV) of 99.8% (880 of 882) and a positive predictive value (PPV) of 95.2% (40 of 42, both intraductal papillomas). Core biopsy was performed in 178 lesions, mostly for indefinite cytology. Core biopsy was definitive in 79.8% cases (57% benign 22% malignant) with a PPV of 100% and NPV of 99.0%. In experienced hands FNAB is an accurate first line diagnostic modality for the assessment of 3B screen-detected discrete masses, providing definitive results in 80.5% of cases. When used as a second line modality, core biopsy had a similarly high rate of definitive diagnosis at 79.8%. The stepwise approach to the use of FNAB and core biopsy would reduce substantially the proportion of cases requiring surgical diagnostic biopsy. Given the low probability of malignancy and the imperative to limit the morbidity associated with cancer screening, the demonstration of the reliability of FNAB as a minimally invasive but highly accurate test for this particular subset of screen-detected lesions has significant clinical utility.

Discrete, circumscribed masses constitute one of the most common groups of lesions encountered during mammographic screening for breast cancer. Such lesions are encountered in approximately 8% of all screening mammograms (Stomper *et al*, 1991) and most are benign (Pisano, 1997).

In the setting of mammographic screening for breast cancer, lesions with characteristically benign mammographic features do not require further assessment. When the mammographic features are not entirely typical of benign lesions, recall for further imaging assessment is indicated (Sickles, 1997). The use of specific imaging techniques, such as magnification views, spot compression and sonography enable radiologists to diagnose cystic lesions and visualise lesions’ contours better, thus determining the benign nature of a high proportion of such lesions with confidence (Kerlikowske *et al*, 2005). However, some cases remain indeterminate despite the additional imaging. Several options are available as the next steps in the management of such lesions. These include surgical excision, core biopsy, fine needle aspiration biopsy or mammographic surveillance.

In the setting of population based screening for breast cancer, where most screen-detected lesions are likely to be benign, the aim to minimise the morbidity associated with cancer screening requires that the use of surgical biopsy be strictly limited to those few cases where imaging and needle biopsy techniques cannot determine the nature of the lesion. Apart from the desire to minimise surgery for benign disease, the large case volume discourages widespread use of open biopsy, and to a lesser extent core biopsy, for pragmatic and economic reasons. The strategy of short-term mammographic follow up is supported by long-term follow up data (Sickles, 1991, 1994; Varas *et al*, 1992). However, since delay in the diagnosis of breast cancer is a source of anxiety for women and their doctors, some clinicians may be uncomfortable with this approach. In this regard, Sickles notes that while patients are initially enthusiastic about the option of short-term mammographic follow up, compliance with the full surveillance protocol was only 45% (Sickles, 1991, 1997).

Fine needle aspiration biopsy (FNAB) is a cost-effective method of investigating breast lesions. In experienced hands and for appropriately selected lesions this test is clinically useful and reliable (Farshid and Downey, 2005). In our programme we use FNAB as a first line diagnostic modality for the assessment of category 3 mass lesions, supplemented where necessary, by core biopsy and surgical biopsy. In the light of the increasing use of core biopsy in preference to FNAB by other programmes, we wished to establish the reliability of benign cytology for this specific category of screen-detected masses through review of long-term outcome data.

## MATERIALS AND METHODS

### The design of our breast cancer-screening programme

BreastScreen South Australia is part of a national breast cancer screening programme and has been accredited to provide this service since 1991. The design of this programme has been described previously (Farshid and Rush, 2004). In brief, asymptomatic women aged 50–69 years are invited to participate at 1or 2 yearly intervals, depending on family history. Two radiologists read two view screening mammograms independently. A third reader arbitrates discordant results. Using the Tabar 5-tier grading scheme (Tabar and Dean, 1983), lesions are assigned to one of five radiologic grades: grade 1, normal; grade 2, benign; grade 3, indeterminate/equivocal, grade 4, suspicious for malignancy and grade 5, radiologically malignant. Clients with grade 1 or 2 lesions are ‘cleared’ and are invited to return for re-screening in 1–2 years. Lesions graded 3 and above are recalled for further assessment. Based on work up mammography and, in most cases, ultrasound examination, the lesion is re-graded and biopsy is performed on those lesions that have not been cleared. Fine needle aspiration (FNAB) has been the initial method of biopsy. More recently, core biopsy has been adopted as the initial method of evaluation of most microcalcifications.

The cytologic smears are reported immediately and are classified into one of five diagnostic categories, based on published guidelines (NHSBSP, 2001). The cytologic categories are: unsatisfactory, benign, atypical/indeterminate, suspicious and positive. Protocols are in place for the further management of each combination of imaging and cytologic findings. Lesions with concordant benign findings (grade 3 with benign cytology) are cleared and are invited for re-screening in 2 years. We liaise with the primary care providers and the State Cancer Registry and are required to be notified of any interval cancers diagnosed in our clients. Lesions with concordant malignant findings (grade 5 with positive cytology) are referred for treatment. Until July 2003 this was also the case for grade 4 lesions with positive cytology. Since then we have required core biopsy confirmation of these diagnoses. Core biopsy may also be performed on grade 5 lesions to clarify the nature of the carcinoma. Lesions with discordant or indefinite FNAB results are assessed by core biopsy. All positive cytology is re-read the next day by an independent second pathologist. We obtain the final histology for all surgical specimens of our clients. All data are continuously audited and prospectively entered into an electronic database.

Our National Accreditation Standards (BreastScreenAustralia, 2005) require that we be notified by the State Cancer Registry of any cancers diagnosed in our clients within 27 months of a negative screen. In addition, breast surgeons and general practitioners also notify us of any breast cancer diagnoses in our clients.

### Study design

The procedures followed in this study were in accordance with our institutional ethical standards and the research was approved by the appropriate institutional review committees.

Discrete solid masses without definite malignant features but with some deviation from the benign criteria are categorised as grade 3B and form the study cases. This is a modification of the Nottingham grading scheme for equivocal lesions, with the ‘B’ referring to the requirement for demonstration of benign epithelial cells on cytology before recommending routine recall. We searched our electronic archives for lesions screened during the period January 1996 to December 2005 in which the dominant radiologic abnormality was coded as a discrete mass, graded radiologically as 3B. For each lesion we tabulated patient demographics, lesion size, biopsy methods and outcome. For lesions that were not excised we obtained follow up information by tracking the clients in our own database during their subsequent screening visits and through the State Cancer Registry.

### Radiologic characteristics

Lesions we regard as likely to be benign have the following characteristics on screening mammograms: sharp margins, low density (but higher than the density of fat), smoothly rounded contours and usually solitary. Lesions with characteristically benign mammographic features, such as typical intramammary nodes, hamartomas, lipomas, oil cysts and typical fibroadenomas are usually not recalled for further assessment. When the imaging features are not typical of a benign lesion but still suggest a probably benign mass, the lesion is graded as 3B and recall for additional imaging is required.

For solid circumscribed masses magnification views, and if necessary, spot compression views are performed. These additional imaging studies assist in excluding malignancy in many cases.

Sonography is particularly useful in distinguishing between solid masses and simple cysts but also has high discriminative value in separating benign solid masses from invasive carcinoma (Stavros *et al*, 1995; Skaane and Engedal, 1998; Chao *et al*, 1999; Chen *et al*, 2004).

## RESULTS

### Case selection

For the time period between January 1996 to December 2005 there are 1183 lesions in our database in which the dominant screen detected abnormality is classified as a category 3B discrete mass.

### Demographic data

The number of such cases assessed each year ranged from 61 to 169 lesions per year, with a mean of 118.3 lesions per year. In line with the general expansion of our programme, there has been a steady increase in the number of these lesions assessed over time. In relative terms, they constitute 24.4% of all lesions biopsied. The mean age of the clients was 55.1 years (std 8.8 years, range 40–86 years). Eighty women (6.8%) gave a very strong family history of breast cancer, 1103 women did not (93.2%). In 387 lesions (32.7%) the client had been using hormone replacement therapy (HRT). 794 women had not used HRT (67.1%). This information is unavailable in two cases.

Five hundred and thirty eight lesions (45.5%) were detected in the first round of screening and the remainder in subsequent rounds (rounds 2–12). The lesion was located in the right breast in 48.1% of cases and in the left breast in 51.9%.

### Imaging features

**Figure 1:**
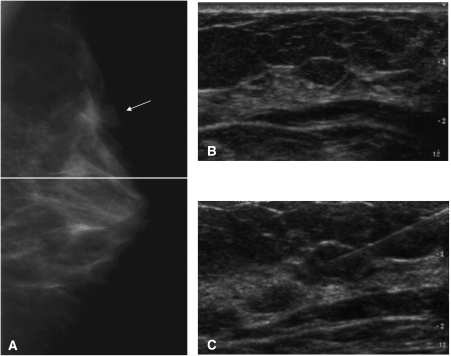
Typical imaging features of a probably benign, circumscribed, discrete mass, graded 3B in our programme. (**A**) Mammogram, left lateral view, with the arrow pointing to a circumscribed mass. (**B**) Breast ultrasound of the lesion, showing a circumscribed oblong mass. (**C**) Ultrasound image, documenting the presence of the biopsy needle within the lesion.

In 1001 lesions (84.6% of cases) there was only one lesion. In the remaining 182 lesions (15.4%) a second lesion was also present requiring assessment. By definition, this other lesion was radiologically of grade 3 or higher. The imaging features of a typical discrete mass included in this study are depicted in Figure 1. This was shown to be a fibroadenoma. The location of the mass was specified in all but five cases. The upper outer quadrant was the most common site (43.5%), followed by the central region (17.5%), upper inner quadrant (15.4%), lower inner quadrant (11.7%), axillary tail (0.8%) and nipple (0.7%). The mean diameter was 13.3 mm (std 8.3 mm, range 2–120 mm).

### Final outcome

After diagnostic work up, tissue biopsy and long term follow up via two independent means, ninety-eight cases (8.3%) were proven to be malignant. These comprised 66 invasive, 26 *in situ* carcinomas and six other neoplasms. This latter group included one primary leiomyosarcoma, three cases of lymphoma, one case of metastatic renal cell carcinoma and one intramammary node with metastatic breast cancer. Of the remaining lesions 1083 have been established to be non-malignant (91.5%). A final diagnosis was not specified in the database for two lesions.

### Cytology

**Figure 2:**
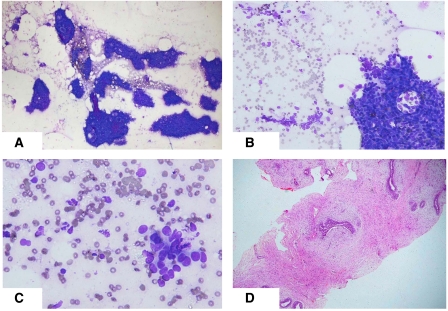
Biopsy features of a case shown to be a fibroadenoma. (**A**) Low power photomicrograph of a fine needle aspiration biopsy of a discrete solid mass, showing mono-layered sheets of cohesive cells in a background of bipolar cells. (**B**) Cohesive sheet of cells together with smaller cell clusters with a tendency to dissociation. (**C**) Higher power view showing small, loosely aggregated cells with mild nuclear enlargement and variability. In view of these features, core biopsy was performed. (**D**) Core biopsy demonstrating features of a fibroadenoma with mild hyperplasia in some ducts.

**Figure 3:**
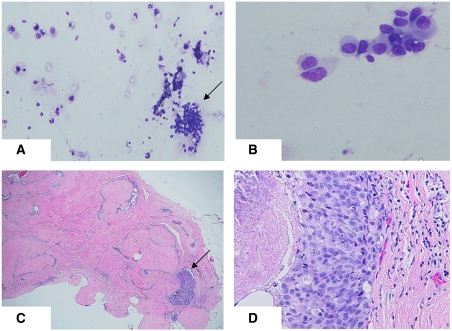
Biopsy features of a fibroadenoma accompanied by a focus of high grade DCIS. (**A**) Low power view of a smear showing cohesive clusters of orderly cells (arrow), together with a dispersed population of large, atypical cells (left side of picture). (**B**) Higher power view of a small cluster of atypical cells, showing nuclear enlargement, variability and irregularity of nuclear membranes. (**C**) Core biopsy showing a fibroadenoma accompanied by one focus of high-grade DCIS (arrow). (**D**) Higher power view of the area of DCIS arising in a fibroadenoma. High-grade nuclear features and comedo necrosis are evident.

**Figure 4:**
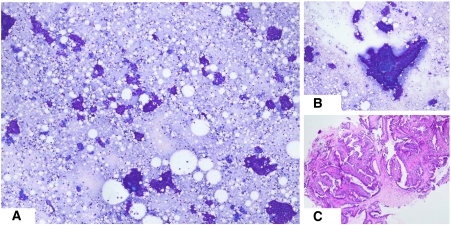
Biopsy features of an intraductal papilloma. (**A**) Low power photomicrograph showing a highly cellular smear, composed of variable sized, cohesive clusters of orderly cells, numerous bare bipolar cells and some dissociated cells. (**B**) Higher power view of a cellular cluster showing branching fibrovascular cores, suggestive of a papillary lesion. (**C**) Core biopsy confirming an intraductal papilloma.

Fine needle aspiration biopsy was performed in 1149 cases (97.1%). Cytology was interpreted as benign in 882 cases (76.8%), atypical in 125 (10.9%), suspicious in 44 (3.9%), malignant in 43 lesions (3.7%) and inadequate in 55 (4.8%). FNAB was not performed in 32 cases, mostly because core biopsy was used as the initial method of assessment at the discretion of the assessment team. The morphology of three different lesions in this study, each assessed by FNAB and core biopsy, are depicted in Figures 2, 3 and 4.

The correlation of the cytologic results against the final diagnosis is given in Table 1. Eight hundred and eighty of 882 (99.8%) lesions with benign cytology were benign. The two false-negative cases were both ductal carcinoma in situ (DCIS). In one case the cytology was interpreted as benign but some papillary features were noted. Since we recommend excision of papillary lesions, open biopsy was performed. This lesion was found to be a papillary and cribriform DCIS, eventually requiring mastectomy for complete excision. The second case had benign cytology. On excision for treatment of an adjoining higher grade lesion, the category 3 mass was found to comprise an area of fibrocystic change with a small focus of high grade DCIS. It is likely that the malignant focus in this mass was not sampled by FNAB.

In all 40 of 42 lesions (95.2%) with malignant cytology were malignant, amounting to a false-positive rate of 4.8%. The cases with false-positive cytology were both intraductal papillomata. The likelihood of malignancy was 18.4% among lesions with atypical cytology and 63.6% for lesions with suspicious cytology. Among lesions that were ultimately proven to be benign, but had initially produced suspicious cytology, fibroadenoma was the commonest lesion (six cases), followed by reactive lymph nodes (three lesions), and one case of each of papilloma, hamartoma, usual ductal hyperplasia and atypical ductal hyperplasia.

### Pattern of use of core biopsy

Core biopsy was used in 178 lesions in this series (15%). In 158 cases this followed FNAB. Core biopsy was required due to indefinite cytology in 126 cases comprising 35 cases with inadequate cytology, 69 with atypical cytology, and 22 with suspicious cytology. In 32 other cases core biopsy was performed to confirm positive cytology results (19 cases), or benign cytology results (13 cases) at the discretion of the assessment team. Core biopsy was the primary biopsy modality in remaining 20 cases.

Table 2 shows the correlation between the core biopsy results and the final diagnosis. Core biopsy provided a definitive benign or malignant diagnosis in 79.8% of cases. One of 102 cases with benign core biopsy had positive cytology and was ultimately found to be malignant at surgery, the core being non-representative. The negative predictive value (NPV) of a benign core biopsy diagnosis was 99.0%. The positive predictive value (PPV) of a malignant core biopsy was 100%. The few cases with suspicious core biopsy findings were found to be malignant in 71.4% of cases and those with atypia on core biopsy were malignant in 7.1% of cases.

In 158 cases core biopsy followed cytologic assessment. Table 3 shows these results. When core biopsy followed insufficient, atypical or suspicious FNAB, it established a definitive benign or malignant diagnosis in 77.8% of cases (98 of 126 cases). Core biopsy was positive for malignancy in 2.9% cases with inadequate cytology, 11.6% cases with atypical cytology, 50% of those following suspicious cytology and in 89.5% after positive cytology. In 19 cases with positive cytology, core biopsy was reported as malignant in 17 of these cases, suspicious in one case and benign in one case. Ultimately, all 19 lesions were indeed malignant, 17 invasive and two *in situ* carcinomas. Core biopsy was therefore helpful in distinguishing the two cases of DCIS from invasive cancers. The one case with benign core biopsy was a false negative due to a sampling problem. In no case with a prior benign cytologic diagnosis did core biopsy show malignancy.

Overall, the core biopsy results were definitive in 79.8% of the cases: this included definite benign results in 57.3% of cases and a definite malignant diagnosis in 22.5%. The remaining 20.2% of cases had indefinite core biopsy results, including those with atypical, suspicious or nonrepresentative cores, all of which required further investigation.

### Cases with final histologic diagnoses

Cases assessed with cytology are categorised according to the 5-tiered diagnostic system described above, but a specific tissue diagnosis is usually not possible in the majority of cases. A specific histologic diagnosis is available in the database in 271 lesions, principally from surgical specimens.

As shown in Table 4 malignancy was documented in 98 of the discrete screen-detected masses in this series. Ductal carcinoma of no special type was the most common carcinoma, followed by mucinous carcinoma. Two tubular carcinomas and one case each of medullary and lobular carcinoma also presented in this manner. Among the malignant lesions there were three cases of lymphoma, one primary breast leimyosarcoma, one metastatic deposit of renal cell carcinoma to the breast parenchyma and one case of an intramammary node bearing metastatic breast cancer.

Among the 66 invasive cancers, infiltrating ductal carcinoma of no special type constituted 74.2% of the lesions, while special type cancers together accounted for 25.8%. Grade I tumours were the most frequent cancer, accounting for 49.2% of the cases. Only 17.5% of the cancers were grade 3, and the remaining 33.3% were grade 2. Grade was not specified for three cases.

Of the 25 DCIS cases that presented as a discrete solid mass, only four were of high grade. Many of the DCIS cases in this series had a papillary component.

As we do not advise surgery for the vast majority of lesions found to be benign on biopsy, a specific histologic diagnosis is available in only a small proportion of these cases. As shown in Table 4, fibroadenoma was the most common benign diagnosis, followed by intraductal papilloma, fibrocystic changes, including ductal hyperplasia and inspissated cysts.

Phyllodes tumour was diagnosed through pathological assessment of the excised mass in seven lesions. Six of these were of the benign subtype and one case was a borderline lesion. There were no instances of malignant phyllodes tumour among this series of screen detected discrete category 3 mass lesions.

### Further follow-up

The records of the State Cancer Registry show that there was one interval cancer diagnosed within 27 months of the screening round of interest and that cancer affected the contralateral breast.

Tracking the clients with non-malignant findings during their subsequent rounds of screening showed that 781 of the 920 (84.9%) eligible women returned for subsequent rounds of screening. The mean period of follow-up was 54 months, (range 10.5–130.3 months). During this time seven women developed breast cancer, five invasive and two DCIS. As can be seen in Table 5, in seven of eight cases these cancers either affected the opposite breast or a different quadrant than the index lesion. The final case was a benign complex cyst on FNAB. Six years later microcalcifications were detected in the same quadrant during screening mammography. These were shown to be associated with DCIS of intermediate grade.

## DISCUSSION

The aim of population-based screening for breast cancer is the reduction in mortality from breast cancer through early detection, achieved with minimal morbidity (BreastScreenAustralia, 2005). Because of its contribution to the morbidity and anxiety associated with cancer screening, the benign biopsy rate is one of the key performance indices for screening programmes. BreastScreen Australia's National Accreditation Standards mandate that the use of open biopsies for non-malignant lesions not exceed 0.35% for women having their first screen and 0.16% for subsequent rounds of screening. Discrete rounded masses are among the most common group of abnormalities detected during screening mammography. Stomper *et al* (1991) found well-circumscribed nodules in 13.3% of 1500 screening mammograms. Even when lesions with features of benign intramammary nodes were excluded, such lesions were found in 6.0% of screening mammograms. Many lesions with pathognomonically benign imaging features on screening mammograms are not assessed further, and a high proportion of the remaining lesions can be established to be benign through the use of mammographic work up and sonography (Stavros *et al*, 1995; Skaane and Engedal, 1998; Chao *et al*, 1999; Chen *et al*, 2004). Lesions that remain indeterminate despite the additional imaging still represent a sizable group, particularly if they were to be assessed by surgical biopsy. In our programme such lesions comprise 24.4% of all screen-detected lesions requiring tissue diagnosis. Since these lesions are likely to be benign, careful case selection for open biopsy is required to minimise the benign biopsy rate.

A variety of approaches are utilised for establishing the nature of these indeterminate, but still probably benign lesions. Depending on local expertise and conventions, short interval mammographic surveillance, fine needle aspiration biopsy, core biopsy and open biopsy have been advocated.

The attraction of mammographic surveillance is that the morbidity and costs associated with screening are minimised, particularly when the only available alternative is open biopsy. Sickles has reported the results of a prospective study of 1403 such women with periodic follow up for at least 36 months. During this time 1.4% of the cohort recommended for mammographic surveillance were diagnosed with invasive carcinoma, almost all detected as a result of interval mammographic change or by becoming palpable (Sickles, 1994). A significantly higher likelihood of malignancy was not found among circumscribed masses in that study where the lesion diameter was in the 10–15 mm range or where the woman was older than 50 years, as cancer was diagnosed in 1.6 and 1.7% of the cases respectively. Such data provide some justification for mammographic surveillance as a plausible method of dealing with mammographically benign mass lesions. However, in Sickles's study only 68.3% complied with the follow-up regimen to 36–43 months. This significant attrition rate, when coupled with the fact that delay in the diagnosis of malignancy is one of the chief factors in medico-legal disputes concerning breast disease causes many clinicians to be uncomfortable with the watchful waiting approach.

### Cytology as a first line diagnostic modality for discrete rounded screen-detected lesions

In our programme a tiered approach to biopsy is applied, using a combination of FNAB and core biopsy with referral of highly selected cases for surgical excision. Breast cytology requires a high level of expertise but our data demonstrate that in experienced hands FNAB is a reliable first line diagnostic modality for assessment of discrete, probably benign masses. Benign cytology had a negative predictive value of 99.8% and positive cytology results had a positive predictive value of 95.2%. The NPV of benign cytology compares favourably with the 99.0% NPV of core biopsy, although cases were selected for core biopsy because they had already proven challenging on cytology.

Since a definite benign cytologic diagnosis has a rather high NPV for category 3 masses, we do not advocate further diagnostic tests for this group of clients. They are recommended to return for their next round of screening, usually in 2 years. This approach thus obviates the need for further, more invasive investigations in 76.8% of clients. The PPV of cytology for this group of lesions is also high, but at 95.2%, it is not sufficiently reliable for definitive surgical management (Farshid and Downey, 2005). Our current protocols therefore require confirmation of malignancy by core biopsy in category 3 masses with positive cytology.

It is noteworthy that only 4.7% of the 1147 lesions assessed by FNAB produced an inadequate sample. This is even lower than the 8.7% rate of inadequacy for the entire group of lesions (Farshid and Downey, 2005). The high rates of inadequate samples of FNAB, ranging up to 58.7% in some reports has contributed to discouraging the use of this technique (Ibrahim *et al*, 2001). We believe that the presence of on-site pathologists is key in this achievement, as samples can be examined immediately and re-aspiration may be undertaken if necessary. It is also a reflection of having a team of radiologists experienced in sampling the lesions.

### The value of core biopsy

As well as requiring confirmation of malignancy in discrete mass lesions with positive cytology, lesions with indefinite cytology, inclusive of those with atypical, suspicious and non-diagnostic results, require core biopsy assessment to establish their nature. In this series, these cases together comprise only 23.1% of all probably benign mass lesions. Thus, theoretically the use of cytology limits the morbidity and costs associated with core biopsy to this subset of clients. In this series, 15% of the lesions were assessed by core biopsy, mostly due to inadequate, indefinite or discordant cytology results. The difference between this figure and the 23.1% prediction is due to the fact that our protocols have evolved over the time period under study to limit the use of open biopsy. Furthermore, some flexibility in the use of open biopsy is permitted, to accommodate specific clinical situations.

When used for the above indications, core biopsy was a useful second line diagnostic modality, resulting in a definitive diagnosis in 79.8% of the cases and leaving only 3.0% of the total cases requiring further assessment, mostly by open biopsy. Unlike cytology, there were no false positive diagnoses with core biopsy, resulting in a PPV of 100%. Core biopsy was also useful in discriminating between *in situ* and invasive cancers. We note that the NPV of benign core biopsy was 99.0%, whereas that of benign FNAB was 99.8%. Far fewer cases were subjected to core biopsy, such that the one false-negative case from sampling error had a large effect on the NPV. Nevertheless, this case points out another advantage of using more than one biopsy technique. The cytology in this case was already positive, casting doubt on the representativeness of a benign core biopsy sample. We therefore view the two techniques as complementary.

With the stepwise approach to the use of the available diagnostic techniques, cytology reduces the proportion of cases requiring core biopsy to 23.1%, and the open biopsy rate falls to 3% when following core biopsy. These figures represent significant reductions in morbidity and cost, particularly since the diagnostic accuracy of the assessment process has not been compromised.

### Long-term follow up

One of the most important aspects of this study is the long-term follow up data presented. Alignment of our client database with the State Cancer Registry records reassures us that none of the clients discharged for routine recall, developed an interval cancer attributable to the lesion in question in the 27 months after assessment of their discrete mass lesion. Furthermore, at least 84.9% of eligible clients have returned for subsequent rounds of screening and with a mean follow up of 54.5 months (R 10.5–130.3 months) we have established that none of the lesions recommended for routine re-screening were malignant. This additional follow-up is crucial in bolstering confidence in the results of our combined clinical, radiologic and cytologic assessment of these lesions.

### The rarity of phyllodes tumour

Fibroadenoma is one of the most common lesions presenting as a discrete mass on screening mammograms. One of the sources of concern in the non-surgical management of fibroepithelial lesions is the overlap in the imaging and cytology, and to a lesser extent core biopsy, features of fibroadenoma and phyllodes tumour. This may lead to surgery even when biopsy suggests fibroadenoma. Our data demonstrate that in the setting of a population based breast cancer-screening programme, most such surgery would be for fibroadenomas. Only seven phyllodes tumors were diagnosed, constituting 0.6% of the total group, and while one case was designated borderline, none of the lesions were malignant. It would appear that malignant phyllodes tumour either occurs rarely in the screening target age group but when it does occur, its imaging features are other than a discrete rounded mass.

### Nature of lesions

Malignant lesions were diagnosed in 8.3% of the cases presenting as discrete screen-detected masses. Invasive ductal carcinoma of no special type constituted the largest subtype, but special type cancers, including mucinous and medullary carcinomas, together comprised 25.8% of the invasive cancers. In line with most screen-detected cancers, the tumours were overwhelmingly of grade I, and 17.5% were classified as grade III.

Of interest was the presentation of DCIS as a discrete rounded mass in 25 cases. This is an unusual but well documented presentation for DCIS (Farshid *et al*, 2007). Only four were of high grade, while many had a papillary component. This is in contrast with the typical presentation of screen detected DCIS, which is as microcalcifications and histologically is associated with comedo-necrosis and high nuclear grade.

A metastatic deposit of renal cell carcinoma, metastatic breast cancer to a lymph node, a primary breast leiomyosarcoma and lymphoma were among the non-epithelial malignancies that produced discrete circumscribed masses.

### Safeguards

Certain non-malignant lesions produce atypical features on cytology and are a potential source of false-positive cytologic diagnoses. Papillary lesions, fibroadenomas, ductal proliferative processes, intra-mammary nodes and hamartomas were among such lesions in this series. Papillary lesions are a particular challenge since they may produce smears of abundant cellularity with dissociation and significant cytologic atypia. The demonstration of branching fibrovascular cores, anatomical borders in some cell groups and palisades of cells provide important clues to the papillary nature of the lesion (Orell and Miliauskas, 2005). A feature that we find particularly useful in limiting false-positive diagnoses is the requirement for the absence of benign epithelial groups before a definite malignant cytologic diagnosis is made (Orell, 1999). If benign groups are present, the category of suspicious for malignancy is used, regardless of the degree of atypia in the remainder of the smear. Another safeguard is the independent review of all positive cytologic diagnoses by a second cytopathologist the following day. Finally, in recent years we have required core biopsy confirmation of all grade 3 discrete mass lesions with positive cytology.

## CONCLUSIONS

Discrete mass lesions detected during population-based screening mammography are common and most such lesions are non-malignant. Even among the subset of such lesions recommended for biopsy only 8.3% were malignant. In experienced hands fine needle aspiration biopsy is a reliable first line diagnostic modality for assessment of these lesions. 76.9% of cases were categorised as benign by cytology, with a 99.8% negative predictive value. Core biopsy can then be reserved for the remaining lesions, where it can be expected to assist in establishing the diagnosis in a further 79.8% of cases, thereby limiting substantially the proportion of cases requiring diagnostic open biopsy.

These data on the reliability of benign cytology are rendered even more robust by the fact that long-term follow up through two independent means has demonstrated no missed cancers among the lesions recommended for routine re-screening. This additional follow-up is crucial in bolstering confidence in the results of the triple assessment process for the discrete rounded lesions detected during screening mammography.

## Figures and Tables

**Table 1:** Correlation of cytologic results against final diagnoses for the 1147 lesions assessed by fine needle aspiration biopsy

	**Final diagnostic category**		
	**Malignant**	**Non-malignant**	**Total**
*FNA biopsy results*			
Benign	2 (0.2%)	880 (99.8%)	882 (76.9%)
Atypical	23 (18.4%)	102 (81.6%)	125 (10.9%)
Suspicious	28 (63.6%)	16 (36.4%)	44 (3.8%)
Positive	40 (95.2%)	2 (4.8%)	42 (3.7%)
Insufficient for diagnosis	1 (1.9%)	53 (98.2%)	54 (4.7%)
			
			1147

FNAB=fine needle aspiration biopsy

**Table 2:** Correlation between core biopsy results and final diagnoses for the 178 lesions assessed by core biopsy

	**Final diagnostic category**		
	**Malignant**	**Non-malignant**	**Total**
*Core biopsy results*			
Benign	1 (1.0%)	101 (99.0%)	102 (57.3%)
Atypical	2 (7.1%)	26 (92.9%)	28 (15.7%)
Suspicious	5 (71.4%)	2 (28.6%)	7 (3.9%)
Positive	40 (100%)	0	40 (22.5%)
Insufficient or non-diagnostic	1 (100%)	0	1 (0.6%)
			
			178

**Table 3:** Core biopsy after FNA biopsy

	**Core biopsy results**					
	**Insufficient**	**Benign**	**Atypical**	**Suspicious**	**Positive**	**Total**
*FNA biopsy results*						
Insufficient	0	32 (91.4%)	2 (5.7%)	0	1 (2.9%)	35 (22.2%)
Benign	0	9 (69.2%)	4 (30.8%)	0	0	13 (8.2%)
Atypical	0	41 (59.4%)	18 (26.1%)	2 (2.9%)	8 (11.6%)	69 (43.7%)
Suspicious	1 (4.6%)	5 (22.7%)	1 (4.6%)	4 (18.2%)	11 (50%)	22 (13.9%)
Positive	0	1 (5.3%)	0	1 (5.3%)	17 (89.5%)	19 (12.0%)
						
						158

FNAB=fine needle aspiration biopsy

**Table 4:** Final histology of selected lesions forming discrete masses on screening mammograms

**Final diagnosis of selected lesions forming a discrete screen-detected mass**		
**Diagnosis**	**Number of lesions**	**%**
Invasive ductal carcinoma, NOS	49	18.08
Mucinous carcinoma	13	4.80
Tubular carcinoma	2	0.74
Medullary carcinoma	1	0.37
Lobular carcinoma	1	0.00
DCIS, including papillary	25	9.23
LCIS	1	0.37
Leiomyosarcoma	1	0.37
Lymphoma	3	1.11
Metastasis	1	0.37
Nodal metastasis	1	0.37
ADH	7	2.58
ALH	1	0.37
Cyst	20	7.38
Fibroadenoma	57	21.03
Intraductal papilloma	30	11.07
Fibrocystic changes	28	10.33
Sclerosing Adenosis	4	1.48
Hamartoma	4	1.48
Lymph node, reactive	10	3.69
Mucocele	1	0.37
Myxoma	1	0.37
Myofibroblastoma	1	0.37
Phyllodes, benign	6	2.21
Phyllodes, borderline	1	0.37
Hemangioma	1	0.37
Tubular adenoma	1	0.37
		
Total	271	

ADH=atypical ductal hyperplasia; ALH=atypical lobular hyperplasia; DCIS=ductal carcinoma *in situ*; LCIS=lobular carcinoma *in situ*; NOS=not otherwise specified.

**Table 5:** Cancers diagnosed in subsequent rounds of screening in women with benign discrete mass lesions

			**Subsequent cancers**
**Case**	**Location of cancer**	**Interval**	**Comment**
A	Same quadrant	6 years	3B mass was a cyst that collapsed after aspiration. Cytology benign. 6 years later developed microcalcifications detected by screening and intermediate grade DCIS was diagnosed.
B	Different quadrant	3 years	3B mass in R UIQ was a fibroadenoma by cytology. Subsequent stellate lesion in R UOQ was an invasive grade 3 ductal carcinoma.
C	Different quadrant	5 years	3B lesion was a fibroadenoma, excised from L LIQ. Subsequent stellate mass in L UOQ was a 9 mm invasive grade 1 ductal carcinoma.
D	Other breast	2 years	3B lesion was a reactive node by cytology. 26 months later developed a 16 mm grade 2 invasive ductal carcinoma in the opposite breast. Node negative.
E	Other breast	3 years	3B mass was a cyst. Subsequent tumour was a 20 mm grade 3 invasive cancer with one positive node.
F	Other breast	4 years	3B lesion was a fibroadenoma by cytology. Invasive high-grade cancer developed in the other breast within 23 months of her latest screen.
G	Other breast	4 years	3B mass was a cyst. 4 years later, a 9 mm invasive ductal carcinoma was diagnosed within 24 months of latest screen.
H	Other breast	6 years	3B lesion was a fibroadenoma on cytology. Microcalcifications in the other breast led to the diagnosis of invasive cancer and DCIS.

DCIS=ductal carcinoma *in situ*
